# Endogenous dopamine release under transcranial direct-current stimulation governs enhanced attention: a study with positron emission tomography

**DOI:** 10.1038/s41398-019-0443-4

**Published:** 2019-03-15

**Authors:** Mina Fukai, Tomoyasu Bunai, Tetsu Hirosawa, Mitsuru Kikuchi, Shigeru Ito, Yoshio Minabe, Yasuomi Ouchi

**Affiliations:** 1 Department of Psychiatry and Neurobiology, Graduate School of Medical Science, Kanazawa University, Kanazawa, Japan; 2 Department of Biofunctional Imaging, Preeminent Medical Photonics Education and Research Center, Hamamatsu University School of Medicine, Hamamatsu, Japan; 3 Research Center for Child Mental Development, Kanazawa University, Kanazawa, Japan; 4 Global Strategic Challenge Center, Hamamatsu Photonics KK, Hamamatsu, Japan

## Abstract

Transcranial direct-current stimulation (tDCS) to the dorsolateral prefrontal cortex (DLPFC) has been established as an effective and noninvasive method to modulate cognitive function. Nevertheless, the mechanisms causing those cognitive changes under the tDCS remain largely unknown. We strove to elucidate the cognito-biological relation under the tDCS condition by examining whether the dopamine system activated by tDCS is involved in cognitive changes in human participants, or not. To evaluate the dopamine system, we used [^11^C]-raclopride positron emission tomography (PET) scanning: 20 healthy men underwent two [^11^C]-raclopride PET scans and subsequent neuropsychological tests. One scan was conducted after tDCS to the DLPFC. One was conducted after sham stimulation (control). Results of [^11^C]-raclopride PET measurements demonstrate that tDCS to the DLPFC caused dopamine release in the right ventral striatum. Neuropsychological tests for attentiveness revealed that tDCS to the DLPFC-enhanced participants’ accuracy. Moreover, this effect was correlated significantly with dopamine release. This finding provides clinico-biological evidence, demonstrating that enhancement of dopamine signaling by tDCS in the ventral striatum is associated with attention enhancement.

## Introduction

Over the past few decades, transcranial direct-current stimulation (tDCS) has been widely accepted as a non-invasive brain-stimulation technique. In this procedure, weak direct current is applied through electrodes placed on the scalp to alter cortical activity and excitability^1^. Reportedly, tDCS to the dorsolateral prefrontal cortex (DLPFC) can modulate cognitive functions regarded as functions of the prefrontal cortex, especially attention control^2,3^ and executive function^4^. The mechanisms underlying these cognitive changes that occur under the tDCS remain largely unknown.

Several lines of evidence found in reports of research related to tDCS effectiveness highlight the dopamine system, which contributes to brain stimulation. Administering the dopamine precursor levodopa significantly prolongs tDCS aftereffects^5^. A clinico-genetic study of catechol O-methyltransferase (COMT), an enzyme that breaks down dopamine released into the synaptic gap, revealed that COMT genotypes are associated with tDCS effects^6^. Most recently, a report of a positron emission tomography (PET) study described that tDCS on the DLPFC induces dopamine release in the striatum^7^. Importantly, however, the putative relation between tDCS-induced change in dopaminergic transmission and cognitive enhancement remains untested.

Dopamine has long been regarded as a key neurotransmitter for cognitive control. Specifically, mounting evidence underscores a close connection between the dopamine system and attention. For example, for Parkinson’s disease, a progressive neurodegenerative disease with nigrostriatal and mesocortical dopamine depletion, dopamine system dysfunction is related to attentional impairment^8^. In fact, dopaminergic medication can ameliorate inattention^9^. Similarly, with attention deficit hyperactivity disorder (ADHD), a disorder characterized by severe inattention, reduction in striatal dopamine activity is associated with inattention^10^. It has been demonstrated that inattention is ameliorated by enhancement of dopamine activity^11^.

Executive function refers to a set of cognitive control processes such as working memory, inhibition, shifting and control of attention, planning, reasoning, problem-solving, and impulse control^12^. A relation between the dopamine system and executive function is also known to exist. For example, in patients with Parkinson’s disease, striatal dopamine dysfunction combined with insular dopamine receptor loss is related to decline in executive function^13^. In addition, COMT genotype is related to executive function in patients with schizophrenia^14^ and in healthy individuals from sibling pairs discordant for psychosis^15^.

Until fairly recently, the biological underpinnings of the facilitative effects of tDCS on attentional control and executive function were unknown. From their well-designed study examining 32 healthy men using [^11^C]-raclopride PET in 2018. Fonteneau et al.^7^ reported that tDCS-induced dopamine release in the putamen. Their study demonstrated that tDCS on the DLPFC can increase the level of endogenous dopamine released in the striatum in humans. Regrettably, a more intriguing point lay unexplored in their study: cognitive alteration with respect to dopamine release under the tDCS condition. As described above, the dopamine system is related to attentional control and executive function. For that reason, it can be readily inferred that cognitive tests for attentional control and executive function might be desirable. To test this inference, we specifically examined appropriate parts of the cognitive test and analytic approaches in the current study.

Here, we aimed to elucidate the cognito-biological relevance under the tDCS condition by examining whether the dopamine system activated by tDCS is involved in cognitive changes in human participants, or not. Our hypothesis was the following: tDCS enhances striatal dopamine release, which improves attention and executive function.

## Materials and methods

### Participants

We recruited 20 healthy 20–26-year-old men using poster advertisements from a neighboring university. All participants were right-handed native Japanese with an intelligence quotient (IQ) higher than 70 (mean (SD) IQ, 110.1 (6.9); IQ of 97–120) on the Japanese reading test (JART)^16^. Participants with metal implants inside the body, cranial bone fractures, known history of epilepsy or substance dependence or chronic headache, and prospective participants with a known psychiatric disorder or on central nervous system-acting medications were excluded from the study. Written informed consent was obtained before enrollment. The Ethics Committees of Kanazawa University Hospital and Hamamatsu University School of Medicine approved the methods and procedures used for this study, which were applied in accordance with the Declaration of Helsinki. The study was registered with the University Hospital Medical Information Network Clinical Trials Registry (no. UMIN000020583).

### Sample size calculation

To determine the ideal sample size, we had to have the effect size of tDCS on striatal dopamine release. However, there was no study focused on the topic at that time. Hence, instead, we decided to refer to the effect size of repetitive transcranial magnetic stimulation (rTMS) on striatal dopamine release and adjusted it for tDCS.

We referred to a study in which [^11^C]-raclopride PET was used to measure extracellular dopamine concentration after rTMS on the DLPFC in healthy human subjects^17^. We calculated the effect size to be 3.37 based on the reported result. Here, rTMS produces electrical current flow in the subject’s brain via magnetic field and its temporal and spatial resolution are higher than those of tDCS^18^. Considering the possibility that the effect of rTMS is higher than tDCS, we adjusted the obtained effect size. As there was no study that compared the effect of those head-to-head, we referred to the studies using similar tasks to evaluate the effect. According to those studies, the reported effect size of rTMS on the subject’s performance in a memory task was 2.00^19^ and that of tDCS was 0.76^20^. Using those values, we estimated effect size of tDCS on striatal dopamine release to be 3.37 × 0.76/2.00 = 1.27. We set alpha value to be 0.0005 and beta value to be 0.8, and the ideal sample size was calculated to be 18. For the sample size calculation, we used G∗Power Version 3.1^21^.

### Experimental design

**Fig. 1:**
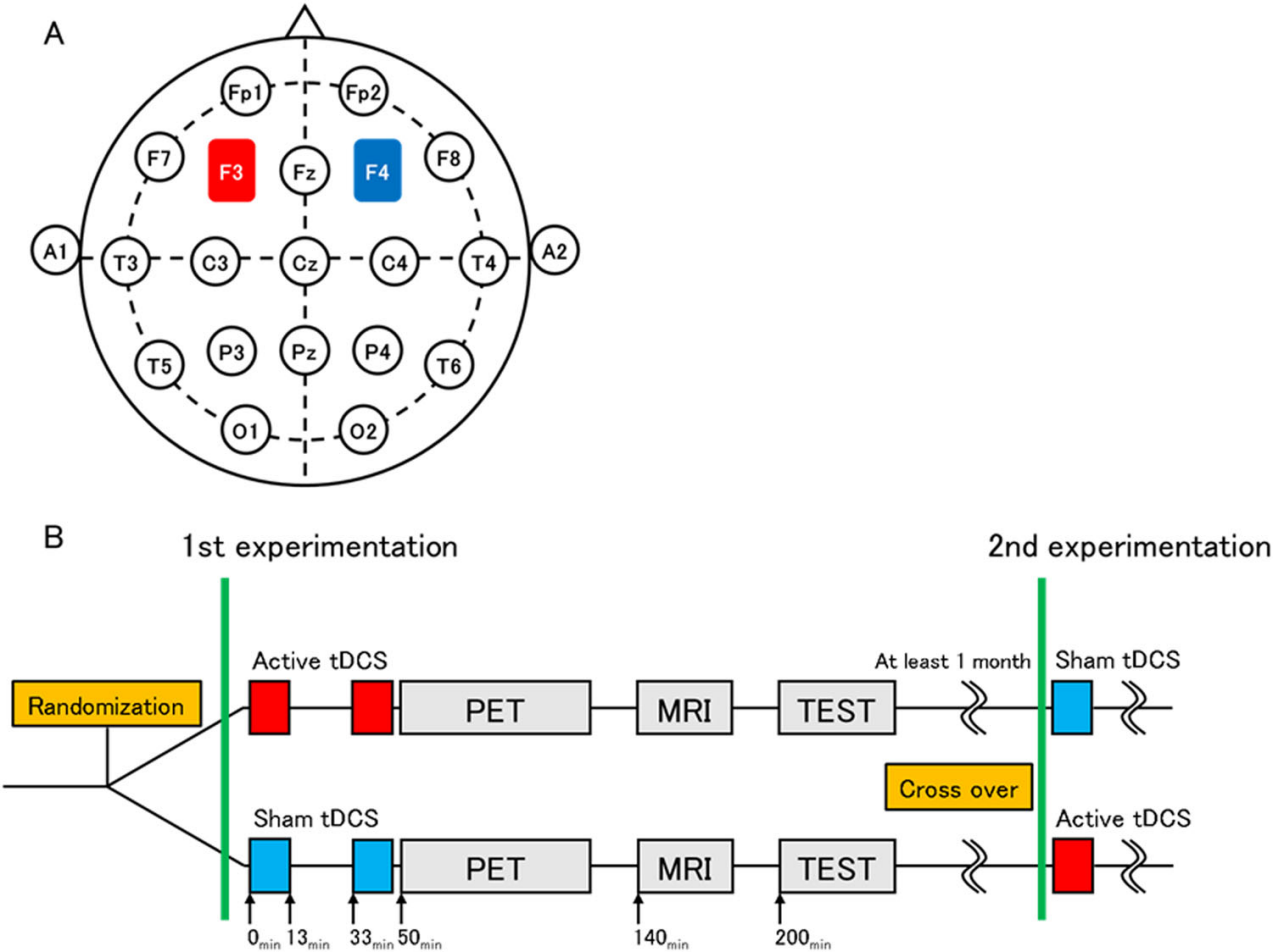
Design of this study The placement of the tDCS electrodes was based on the international 10–20 EEG system: the anode at the F3 (left DLPFC) and the cathode at the F4 (right DLPFC) **a**. The type of stimulation (active or sham) was randomized in a double-blind manner. Thirteen-minutes duration tDCS was applied twice with an interval of 20 min. PET was performed at 50 min after initiation of tDCS, followed by MRI measurement at 140 min and cognitive tests at 200 min, respectively. Participants underwent twice experiments in a crossover fashion at least 1 month apart **b**

This study used a randomized, sham-controlled, double-blind, crossover design. Participants completed two sessions at least 1 month apart to control for carry-over effects. Participants then underwent 26 min of tDCS (either cathodal or sham) on DLPFC, after which PET/magnetic resonance imaging (MRI) and the cognitive function test were performed (Fig. 1).

Randomization was centralized, and a computer-generated list was used in the Innovative Clinical Research Center, Kanazawa University Hospital. The study staff did not have access to the allocation sequence until the end of the study.

### Intervention: tDCS

Via two soaked (NaCl 0.9%) electrodes (35 cm^2^) using a stimulator (DC-Stimulator Plus II; neuroCare Group GmbH, Ilmenau, Germany), participants received either 13 min × 2 of anodal stimulation (2 mA) or sham stimulation to the left hemisphere. In addition, they received 13 min × 2 of active stimulation (2 mA) or sham stimulation to the right hemisphere of the brain. The stimulation type (active or sham) was randomized across sessions and was counterbalanced across participants: 10 completed sham then active; the remaining 10 completed active then sham.

The anode and cathode locations were almost identical to those used in a recent study:^7^ the anode was placed at the center of the electrode over the left DLPFC (F3); the cathode was over the right DLPFC (F4) according to the international 10–20 EEG system, as presented in Fig. 1a. Despite the electrode location similarity, the protocol of tDCS application and PET measurements differed from those earlier reported methods with post-injection stimulation during continuous infusion PET measurement^7^. The tDCS effect is known to continue up to more than 24 h^22,23^. Therefore, we delivered the stimulation before bolus-infusion PET measurements (Fig. 1b). The sham stimulation was applied for 30 s before the PET scan, where some participants were aware of the initial tingling sensation, but none noticed the difference in active or sham stimulation.

### Cognitive evaluation

We used the Cambridge Neuropsychological Test Automated Battery (CANTAB), a semi-automated computer interface for assessing cognitive function. Within CANTAB assessments, participants completed tests evaluating attention control (Reaction time, response to intervention, RTI) as a main behavior target. In addition, as complementary targets, we used a task for working memory (Spatial Working Memory task, SWM).

### Reaction time

In RTI, five circles are presented on the upper screen. Then, a yellow dot will appear in one of the circles. The participant must react as soon as possible, selecting the circle in which the dot appeared. Outcome measures include the reaction time and the accuracy scale. The reaction time measures the participant’s response latency. The accuracy scale is the total number of trials for which the response is correct.

### Spatial working memory task

SWM began with a number of colored squares shown on the screen. The aim of this test is that by selecting the boxes and using a process of elimination, the participant should find one yellow “token” in each of a number of boxes and use them to fill up an empty column on the right-hand side of the screen. The number of boxes can be increased gradually up to a maximum of 12 boxes. Participants are informed that once a “token” is found, that box will not hide another one in that search set. Individual differences in the ability to adopt a consistent search sequence were evaluated and defined as the strategy score, which represents the executive working memory ability^22^.

### PET data acquisition

We performed PET scanning using a high-resolution brain PET scanner (SHR12000; Hamamatsu Photonics K.K., Hamamatsu, Japan) with image resolution of 2.9 × 2.9 × 3.4 mm full-width at half-maximum. A thermoplastic face mask was used to fix the head in the same place during the scans. Then [^11^C]-raclopride examination was conducted of all participants. After a 10-min transmission scan for attenuation correction with a ^68^Ge/^68^Ga source, serial emission scans (time frames, 6 × 10 s, 3 × 20 s, 2 × 60 s, 2 × 180 s, 10 × 300 s) were conducted for 60 min. All participants underwent two scans with an interval of > 1 month between stimulations.

### MRI scanning

To ascertain the areas of the regions of concern for setting regions of interest (ROIs), MRI was applied using a 3 T MRI device (3 T Ingenia; Philips Healthcare, Best, The Netherlands) with the following acquisition parameters: three-dimensional mode sampling, TR shortest (6 ms) and TE shortest (2.7 ms), 8° flip angle, 0.9 × 0.9 × 0.9 mm voxel size, 210 slices). Our mobile PET gantry enabled us to reconstruct PET images parallel to the estimated intercommissural line without reslicing. Using this approach, we were able to allocate ROIs on the target regions of the original PET images.

### Image data analyses

The binding potential (BP_ND_) of [^11^C]-raclopride in each region was estimated using non-invasive Logan plot analysis with software (PMOD 3.5; PMOD Technologies LLC, Zurich, Switzerland). As described in earlier reports of the literature^24,25^, the putamen was selected as the target region. The cerebellum was selected as the reference region for [^11^C]-raclopride. Time–activity curves (TACs) were extracted for each region under active and sham stimulation conditions.

### SPM analysis

To identify the dopaminergic projection regions after active stimulation conditions, a voxel-wise analytic method was performed with software (Statistical Parametric Mapping 8, SPM8; Wellcome Department of Imaging Neurosciences, London, UK) running on Matlab 8.6 (The MathWorks Inc., Natick, MA, USA). All [^11^C]-raclopride early accumulation (perfusion) images were first normalized to the Montreal Neurological Institute (MNI) 152 standard space. Then, with the obtained transformation parameters, the parametric BP_ND_ images were spatially normalized and smoothed with an isotropic Gaussian kernel of 6 mm: nearly twice the scanner resolution. Paired *t* tests in SPM8 were applied voxel-wise to examine the brain regions that showed significant reduction in receptor binding (increase in dopamine release) after the active stimulation condition compared with the sham stimulation condition. The intensity threshold was inferred as *p* < 0.001 uncorrected for multiple comparisons at the voxel level over 10 contiguous voxels because areas of interest were expected a priori^16^. The final SPM images were then generated by masking out the extra-striatal white matter. The variation of BP_ND_ of [^11^C]raclopride PET data were not different between two groups, which allowed further analysis of SPM that was based on the BP_ND_ PET data.

### Statistics for ROI analyses

The ROIs were located bilaterally on the dopaminergic projection regions (the caudate, ventral striatum, and dorsal striatum) displayed on the 5–10 PET slices, which indicates that ROIs from each region contained functional information of the region 9–18-mm-deep in the *z* direction. These ROIs or volumes of interest were transferred automatically onto corresponding [^11^C]-raclopride BP_ND_ parametric images. Regional [^11^C]-raclopride BP_ND_ derived from these ROIs proceeded using PMOD software. Data were analyzed using software (Statistical Package for Social Sciences ver. 23; SPSS Inc., Chicago, IL, USA). Paired *t* tests were applied to compare the levels of [^11^C]-raclopride BP_ND_ in the respective brain regions between active and sham stimulation conditions. For all results, statistical significance was inferred for *p* < 0.05.

### Statistical evaluation for effects of tDCS/[^11^C]-raclopride BP_ND_ on accuracy/reaction times in RTI (specifically examining attentiveness)

With regard to behavior performance, our main objective was twofold: to evaluate the putative relation between the change in dopamine concentration at the striatum and the change in accuracy/reaction time in RTI, and to evaluate the putative effects of tDCS on those measures.

Before applying linear regression, we used regression diagnostics to verify how well our data met the assumptions used for regression analysis. Specifically, we checked the following assumptions: linearity, normality, homogeneity of variance, model specification, influence, and collinearity, and the Gaussian distribution of overall error distribution in each model. In some models, graphical tests suggest the presence of heteroscedasticity in our model. In such cases, we used heteroscedasticity-robust standard errors^26^.

First, we applied paired *t* tests with z-transformation modified data to offset physiological and psychological variations among participants for evaluation of the effects of tDCS on the scores of mean accuracy and standard deviation of reaction time in RTI controlling for age. A larger standard deviation implies that the reaction time is so irregular that participants have difficulty performing tasks efficiently. We applied simple regression analyses to evaluate relations between post-tDCS changes of these two scores and post-tDCS changes in [^11^C]-raclopride BP_ND_ at the ventral striatum.

Second, to confirm relations between dopamine release and cognition in a double-check manner, we applied a linear mixed effects analysis (with robust variance estimation^26^ in cases where it was necessary) to predict the accuracy scores/standard deviation of reaction time in RTI based separately on the value of [^11^C]-raclopride BP_ND_, the type of stimulation (i.e., tDCS vs. sham) controlling for age, and interaction between age and [^11^C]-raclopride BP_ND_. As fixed effects, we entered [^11^C]-raclopride BP_ND_ or a stimulation type (i.e., tDCS vs. sham) and age along with their two-way interaction into the model. As a random effect, we used intercepts for participants. To evaluate the main effect of the value of [^11^C]-raclopride BP_ND_ or the type of stimulation under the existence of the interaction between them, we centered the ages at their means. Statistical significance was inferred for *p* < 0.05.

### Statistical evaluation of tDCS/[^11^C]-raclopride BP_ND_ effects on strategy scores in a SWM (particularly addressing executing function)

As a secondary endpoint, we evaluate the relation between the change in dopamine concentration at the striatum and the change in strategy score in SWM, and the effects of tDCS on those measures. The statistical procedures (paired *t* test and a linear mixed effect analysis) were the same as those described in the previous section, except for those related to cognitive data (strategy scores in SWM).

All statistical analyses were conducted using software (Stata ver. 15.0; StataCorp LLC, College Station, TX, USA).

## Results

### Post-tDCS changes in [^11^C]-raclopride binding

**Fig. 2:**
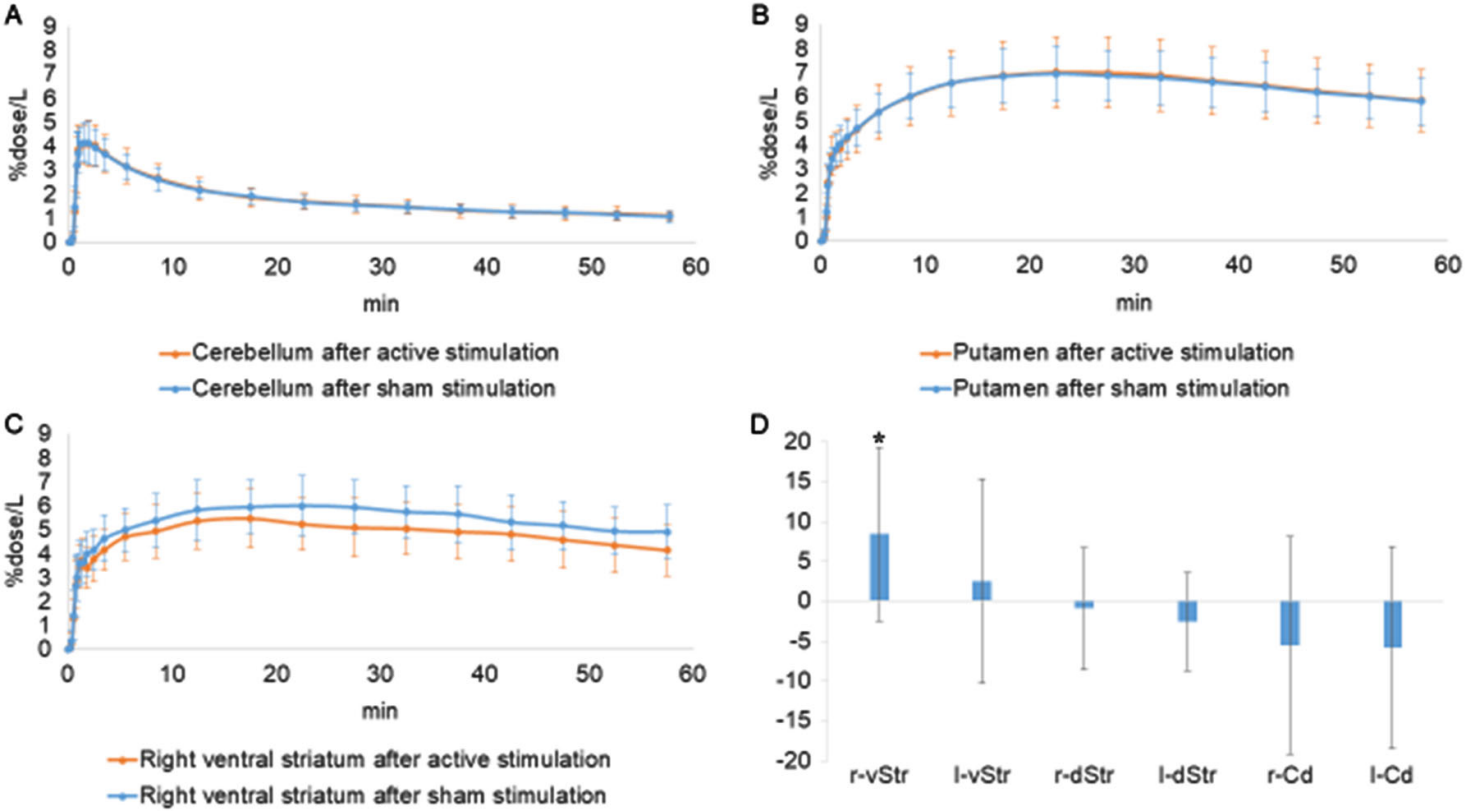
Time–activity curves (TACs) of [11 C]-raclopride No difference was found between TACs from the cerebellum **a** and putamen **b** except for the right ventral striatum **c**. In this region, the magnitude of percentage of reduction in [^11^C]-raclopride binding was found to be higher after active stimulation (*p = 0.002) **d**. The vertical axes show %dose/L. The red curve shows active stimulation. The blue curve shows sham stimulation

**Fig. 3:**
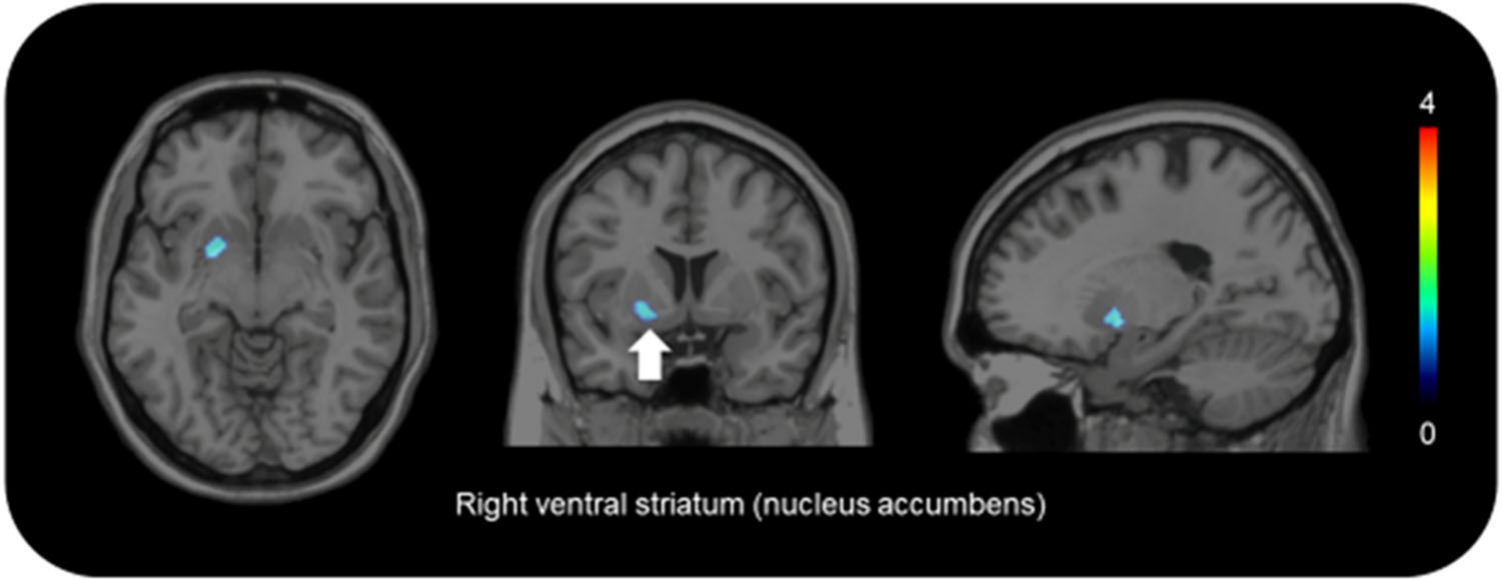
Brain region of increased dopamine release The brain region superimposed on magnetic resonance images shows marked reduction in [^11^C]-raclopride binding after active stimulation. The color bar shows the *t* value

The TACs of [^11^C]-raclopride are presented in Fig. 2. The patterns of TACs from the putamen and cerebellum showed no difference between the stimulation conditions (Fig. 2a, b). Paired *t* tests showed that the concentrations of [^11^C]-raclopride BP_ND_ in the right ventral striatum after active stimulation were significantly lower than after the sham stimulation (*P* < 0.001) (Table 1; Fig. 2d). Consistently, the pattern of TAC of the right ventral striatum after active stimulation showed a gradual decrease after reaching a peak (Fig. 2c). No significant change was found in the binding levels in the caudate and dorsal striatum. Statistical parametric mapping confirmed this result. We found significant reduction in the [^11^C]-raclopride BP_ND_ concentration in the right ventral striatum after active stimulation (Figs. 2d, 3).

**Table 1:** Binding potentials for [11C]-raclopride after active stimulation and sham stimulation

Stimulation	Caudate		Ventral striatum		Dorsal striatum	
	Right	Left	Right	Left	Right	Left
Active	1.74 ± 0.28	1.66 ± 0.28	1.54 ± 0.24	1.66 ± 0.28	2.99 ± 0.31	3.03 ± 0.30
Sham	1.66 ± 0.24	1.58 ± 0.29	1.69 ± 0.22*	1.71 ± 0.25	2.98 ± 0.37	2.96 ± 0.33
%reduction	−5.544186646	−5.77817012	8.417961202	2.489250635	−0.920675852	−2.593258088

Binding potentials for [^11^C]-raclopride after active and sham stimulation: values are expressed as mean ± s.d.; %reduction, mean value of reduction (percentage) in [^11^C]-raclopride binding between active and sham stimulation conditions. *p < 0.001 versus sham stimulation

### Relations between post-tDCS changes in [^11^C]-raclopride BP_ND_ and post-tDCS changes in each task performance after controlling for age

#### (a) accuracy/standard deviation of reaction time in RTI (attentiveness)

**Fig. 4:**
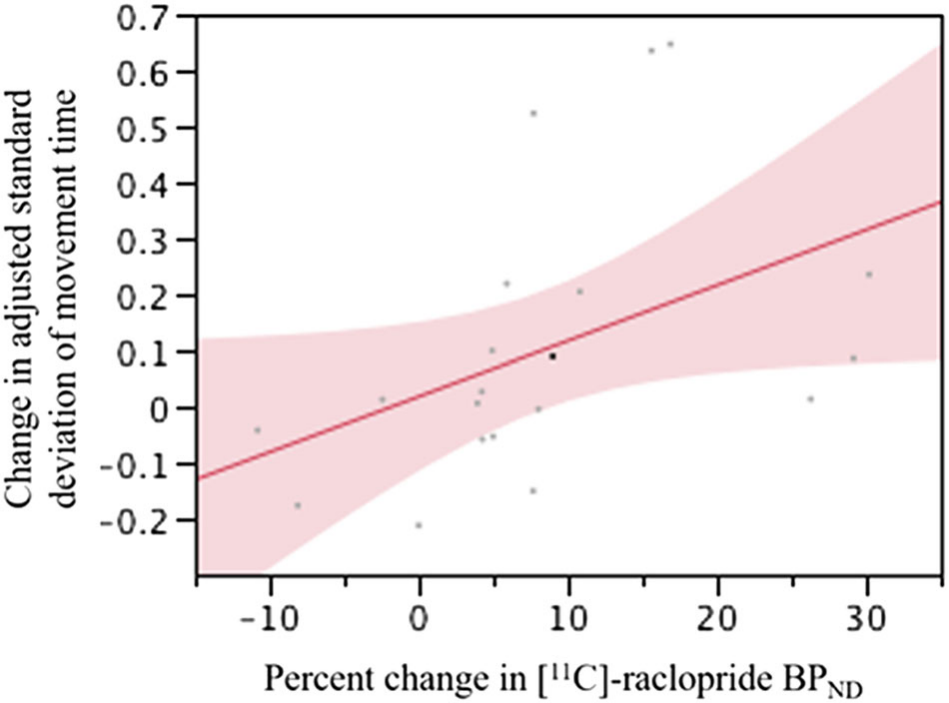
Relation between changes in adjusted standard deviation of reaction time and in [^11^C]-raclopride BP_ND_. BP_ND_, binding potential

Simple regression equations showed no significance for either the accuracy or standard deviation of reaction time in RTI (*F*(1,18) = 0.05, *p* = 0.83, *F*(1,18) = 1.77, *p* = 0.20, respectively). Therefore, we reanalyzed the raw values of accuracy or standard deviation of reaction time that were weighted by each *Z* score to emphasize changes from the baseline^27^. Using this model, we applied regression analyses to ascertain whether changes of these adjusted scores (i.e., raw values multiplied by their respective *Z* scores) correlated, or not, with changes of [^11^C]-raclopride BP_ND_ data, controlling for age. We found a significant regression equation only in the case for the standard deviation of reaction time (*F*(1,18) = 4.5, *p* = 0.048), with *R*^2^ of 0.20. [^11^C]-raclopride BP_ND_ was found to be a significant predictor of the standard deviation of reaction time in RTI (*t* *=* 2.12, *p* = 0.048) (Fig. 4). No significant correlation was found for accuracy (*df* = 19, *t* = 1.58, *p* = 0.130).

#### (b) strategy scores in SWM (executive function)

No significant regression equation was found for the strategy scores (*F*(1,18) = 0.67, *p* = 0.43, *R*^2^ of 0.04). Therefore, [^11^C]-raclopride BP_ND_ was not found to be a significant predictor of the strategy score in SWM (*t* *=* 0.82, *p* = 0.43).

### Linear mixed effect analysis for relations between [^11^C]-raclopride BP_ND_/types of stimulation and each task performance after controlling for age

#### (a) relation between [^11^C]-raclopride BP_ND_ and accuracy/standard deviation of reaction time in RTI

Results show a significant two-way interaction effect ([^11^C]-raclopride BP_ND_ × age) (*z* = −3.35, *p* = 0.001) for accuracy in RTI: main effects for [^11^C]-raclopride BP_ND_ (*z* = −2.59, *p* = 0.009) and age (*z* = −3.73, *p* < 0.001) (Table 2). In addition, we found a significant two-way interaction effect ([^11^C]-raclopride BP_ND_ × age) (*z* = −2.76, *p* = 0.006) for the standard deviation of reaction time: main effects for [^11^C]-raclopride BP_ND_ (*z* = −3.24, *p* = 0.001) and age (*z* = −2.53, *p* < 0.012) (Table 2). These results imply that lower [^11^C]-raclopride BP_ND_ (i.e., a higher concentration of dopamine) at the ventral striatum corresponds to higher accuracy and lower standard deviation of reaction time in RTI after controlling for the interaction term.

**Table 2:** Relations between changes in [^11^C]-raclopride BP_ND_ and changes in accuracy/standard deviation of reaction time for RTI

Variable	Coeff.	SE	*z*	*P* > *z*	95% CI
Accuracy scale					
[^11^C]-raclopride BP_ND_	−0.99	0.38	−2.59	0.009	−1.73 to −0.24
Age	−0.50	0.13	−3.73	<0.001	−0.76 to −0.24
[^11^C]-raclopride BP_ND_ × Age	−0.30	0.09	−3.35	0.001	−0.48 to −0.13
Standard deviation of reaction time					
[^11^C]-raclopride BP_ND_	14.1	4.33	3.24	0.001	5.55–22.6
Age	−2.96	1.17	−2.53	0.012	−5.25 to −0.66
[^11^C]-raclopride BP_ND_ × Age	−2.80	1.01	−2.76	0.006	−4.80 to −0.80

Coeff., regression coefficient; SE, robust standard error; CI, confidence interval; BP_ND_, binding potential

Age and BP_ND_ are centered at their means (i.e., means were subtracted from all scores)

#### (b) effects of tDCS on accuracy/standard deviation of reaction time in RTI

We found a significant two-way interaction effect (stimulation × age) (*z* = 4.41, *p* < 0.001) for accuracy: stimulation (*z* = 2.57, *p* = 0.01) and age (*z* = −5.48, *p* < 0.001) (Table 3), suggesting that tDCS enhanced the accuracy in RTI, after controlling for the interaction term, and that the effect of tDCS varies depending on age.

**Table 3:** Stimulation effects on accuracy/standard deviation of reaction time in RTI

Variable	Coeff.	SE	*z*	*P* > *z*	95% CI
Accuracy scale					
Stimulation (tDCS = 1/Sham = 0)	0.87	0.33	2.57	0.01	0.20–1.52
Age	−0.81	0.15	−5.48	<0.001	−1.11–0.52
Stimulation × age	0.78	0.18	4.41	<0.001	0.43–1.12
Standard deviation of reaction time					
Stimulation (tDCS = 1/Sham = 0)	−7.00	4.04	−1.72	0.086	−14.9–0.97
Age	−3.94	2.25	−1.75	0.081	−8.36–0.48
Stimulation × age	2.55	2.23	1.15	0.252	−1.81–6.93

Coeff., regression coefficient; SE, standard error; CI, confidence interval; tDCS, transcranial direct-current stimulation

Age is centered at its mean (i.e., means were subtracted from all scores)

#### (c) relation between [^11^C]-raclopride BP_ND_ and strategy scores, and effects of tDCS on strategy scores in spatial working memory task

Although significant main effects for stimulation (*z* = −2.93, *p* = 0.003) on strategy scores were found in SWM, no other factor was found to be significant (Table 4). This finding suggests that participants under the tDCS might take more strategic processes to reach a goal. As opposed to the case for accuracy/standard deviation of reaction time, however, the relation between [11C]-raclopride BP_ND_ and strategy scores was not significant.

**Table 4:** Relations between changes in [^11^C]-raclopride BP_ND_/types of stimulation and changes in strategy scores in SWM

Variable	Coeff.	SE	*z*	*P* > *z*	95% CI
[^11^C]-raclopride BP_ND_					
[^11^C]-raclopride BP_ND_	−2.49	1.33	−1.88	0.060	−5.10–0.10
Age	−0.07	0.32	−0.22	0.827	−0.70–0.56
[^11^C]-raclopride BP_ND_ × age	0.3	0.31	0.95	0.340	−0.31–0.92
Stimulation					
Stimulation (tDCS = 1/Sham = 0)	−1.77	0.58	−3.02	0.003	−2.91 to −0.61
Age	−0.05	0.36	0.14	0.886	−0.66–0.76
Stimulation × age	−0.08	0.33	−0.25	0.800	−0.72–0.56

Coeff., regression coefficient; SE, standard error; CI, confidence interval; BP_ND_, binding potential

Age and BP_ND_ are centered at their means (i.e., means were subtracted from all scores)

## Discussion

Our study demonstrated that tDCS caused a significant release of a dopamine in the right ventral striatum in healthy human male participants. The neuropsychological test for attentiveness revealed that tDCS enhanced participants’ accuracy in the RTI task. This effect was correlated significantly with dopamine release. In addition, tDCS enhanced participant’s executive working memory. This effect, however, was not correlated significantly with dopamine release.

As described in the Introduction, dopamine neurotransmission is regarded as playing a crucially important role in the mechanism of the action of tDCS^5–7^. The present report is the first describing a direct relation between dopamine release and cognitive enhancement of tDCS in humans. In fact, the present result is consistent with those of a recent study, demonstrating tDCS-induced dopamine release^7^. In that study, dopamine release was observed in the striatum on the hemisphere contralateral to the anode attachment. The loci in these two studies, however, were slightly different. Although we found a significant increase only in the right ventral striatum, Fonteneau et al., found a significant increase in the right dorsal striatum and the left putamen. Those different results might be attributable to the different stimulation methods used in the respective studies. Participants in our study received 13 min of stimulation twice. By contrast, those in Fonteneau’s study received 20 min of stimulation once, suggesting that single and longer simulation might induce dopamine release over a wider area.

In line with our hypothesis, enhanced attention corresponded to the increased release of dopamine in the striatum. Although the observation merely points to an association, mounting evidence suggests that increased dopamine release in the striatum enhances attention; collectively, that evidence might be sufficient to infer a causal relation. For example, in ADHD, a disorder that manifests hyperactivity/impulsivity and inattention, striatal dopamine activity is well known to be depressed^10^. The depressed striatal dopamine activity contributes to symptoms of inattention^10^_._ Dopaminergic agents ameliorate the inattention^28^. It is noteworthy that, similarly to the mode of stimulation used in our study, anodal tDCS to the left DLPFC improved ADHD symptoms in adolescents and adults^29,30^. Considering these facts, our results confirm that tDCS induces dopamine release in the striatum and confirm that the enhanced dopaminergic transmission in the striatum eventually enhances attention.

Regarding SWM, we observed enhanced strategy scores after tDCS. Unexpectedly, however, the dopamine concentration measured at the ventral striatum was not correlated significantly with the strategy score. Working memory is conceptualized according to a model in which the central executive regulates the modality-specific storage system^31^. Supporting this concept, working memory capacity and executive functioning constructs are reportedly well correlated^32^. The strategy scores in SWM provide a measure of the executive working memory capacity^23^. The present result implies a positive effect of tDCS (for DLPFC) on executive function. This hypothesis closely matches results of a recent meta-analysis elucidating the positive effects of tDCS for tDCS on executive function^4^. Furthermore, no correlation was found between the dopamine concentration at the putamen and the strategy score, presenting the possibility that the positive effect of tDCS on executive function can be mediated by systems other than the dopaminergic: candidates are noradrenergic, serotonergic, and cholinergic neurotransmission systems^33^.

Our tentative conclusion from this study of tDCS-induced release of dopamine and concomitant attentional improvement is applicable to young healthy people. Given that dopaminergic transmission in the striatum and attentional ability are expected to decrease with age^10^, an elderly person might be a good candidate for application of tDCS. Our results are based on data from a small sample of young people of a narrow age range. Therefore, to confirm the effects of tDCS on people from a general population, further study must be conducted with larger samples that include elderly people.

Some limitations of our study must be considered. First, we evaluated cognitive functions for only a short period immediately after the stimulation. Therefore, it is not possible to form any inference about the persistence of aftereffects. Second, the lack of women among the study participants prevents generalization of this conclusion beyond men. Third, possibly because of the small sample size, the statistical threshold with FWE corrections could not be applied in the current SPM analysis. For that reason, further studies must be conducted with a larger sample size.

In conclusion, this report is the first describing a study of an association between tDCS-induced dopamine increase and improvement in attention beyond a speed–accuracy tradeoff using single-session tDCS. This finding provides clinico-biological evidence demonstrating that enhancement of dopamine signaling by tDCS in the ventral striatum is associated with attention enhancement.
